# Selection processes of Arctic seasonal glacier snowpack bacterial communities

**DOI:** 10.1186/s40168-023-01473-6

**Published:** 2023-03-02

**Authors:** Christoph Keuschnig, Timothy M. Vogel, Elena Barbaro, Andrea Spolaor, Krystyna Koziol, Mats P. Björkman, Christian Zdanowicz, Jean-Charles Gallet, Bartłomiej Luks, Rose Layton, Catherine Larose

**Affiliations:** 1 Formerly at Univ Lyon, CNRS, INSA Lyon, Université Claude Bernard Lyon 1, Ecole Centrale de Lyon, Ampère, UMR5005, 69134 Ecully Cedex, France; 2 Currently at Interface Geochemistry, German Research Center for Geosciences, GFZ, Potsdam, Germany; 3 Univ Lyon, CNRS, INSA Lyon, Université Claude Bernard Lyon 1, Ecole Centrale de Lyon, Ampère, UMR5005, 69134 Ecully Cedex, France; 4 Institute of Polar Sciences, ISP-CNR, Via Torino 155, 30170 Venice Mestre, Italy; 5 Department of Environmental Sciences, Informatics and Statistics, Ca’ Foscari University of Venice, Via Torino 155, 30172 Venice, Italy; 6 Department of Environmental Change and Geochemistry, Faculty of Geographical Sciences, the Kazimierz Wielki University in Bydgoszcz, Bydgoszcz, Poland; 7 Department of Earth Sciences, University of Gothenburg, Box 460, SE-40530 Gothenburg, Sweden; 8 Department of Earth Sciences, Uppsala University, Villavägen 16, SE-75236 Uppsala, Sweden; 9 Norwegian Polar Institute, No-9296 Tromsø, Norway; 10 Institute of Geophysics, Polish Academy of Sciences, Księcia Janusza 64, 01-452 Warsaw, Poland

**Keywords:** Microbial ecology, Snow, Arctic, Niche-based selection, Neutral processes

## Abstract

**Background:**

Arctic snowpack microbial communities are continually subject to dynamic chemical and microbial input from the atmosphere. As such, the factors that contribute to structuring their microbial communities are complex and have yet to be completely resolved. These snowpack communities can be used to evaluate whether they fit niche-based or neutral assembly theories.

**Methods:**

We sampled snow from 22 glacier sites on 7 glaciers across Svalbard in April during the maximum snow accumulation period and prior to the melt period to evaluate the factors that drive snowpack metataxonomy. These snowpacks were seasonal, accumulating in early winter on bare ice and firn and completely melting out in autumn. Using a Bayesian fitting strategy to evaluate Hubbell’s Unified Neutral Theory of Biodiversity at multiple sites, we tested for neutrality and defined immigration rates at different taxonomic levels. Bacterial abundance and diversity were measured and the amount of potential ice-nucleating bacteria was calculated. The chemical composition (anions, cations, organic acids) and particulate impurity load (elemental and organic carbon) of the winter and spring snowpack were also characterized. We used these data in addition to geographical information to assess possible niche-based effects on snow microbial communities using multivariate and variable partitioning analysis.

**Results:**

While certain taxonomic signals were found to fit the neutral assembly model, clear evidence of niche-based selection was observed at most sites. Inorganic chemistry was not linked directly to diversity, but helped to identify predominant colonization sources and predict microbial abundance, which was tightly linked to sea spray. Organic acids were the most significant predictors of microbial diversity. At low organic acid concentrations, the snow microbial structure represented the seeding community closely, and evolved away from it at higher organic acid concentrations, with concomitant increases in bacterial numbers.

**Conclusions:**

These results indicate that environmental selection plays a significant role in structuring snow microbial communities and that future studies should focus on activity and growth.

Video Abstract

**Supplementary Information:**

The online version contains supplementary material available at 10.1186/s40168-023-01473-6.

## Background

Microbial diversity is critical for ecosystem functioning; therefore, the underlying mechanisms that drive community structure across space and time are important outstanding puzzles in microbial ecology. Given their size, microorganisms can disperse globally and are found in every ecosystem ever sampled, including snowpacks that can cover over 60 million km^2^ during the northern hemisphere winter [1]. Snow provides a specific physio-chemical environment with nutrients for bacterial and fungal growth and, thus, acts as a habitat for a diverse community of microorganisms [2]. A large body of work has focused on aged perennial snowpacks that have been shown to develop autochthonous snow algal communities (reviewed in [3, 4]), but the study of microbial communities in newly developing seasonal snowpacks outside of the melting period is relatively more recent [5, 6]. Although biological activity in dry seasonal snow remains poorly understood, it is a critical catalyst of organic matter (OM) cycling [7–9]. More recent results have also shown that these snow microorganisms are metabolically active and interact with OM [10–12]. Snowpacks constitute complex ecosystems with diverse microbial inhabitants that interact with their surrounding environment and each other [13–15]. In addition, recent studies have highlighted the continuously ongoing microbial activity, metabolism, and ecological succession in snowpacks [10, 16]. Seasonal terrestrial snowpacks, ones that form over surfaces every year and melt out completely in the autumn, are ideal systems to study the distribution, abundance, and interaction of microorganisms, since they constitute newly formed habitats that are colonized by microorganisms from other sources. These snowpacks can form on bare soil, tundra, frozen lakes, and glaciers and the surfaces upon which they develop likely influence some of the seeding processes. The main seeding sources into freshly developing snow habitats are aerosolized microorganisms that enter the snowpack via wet and dry deposition or colonization from the terrestrial surface upon which the snowpack develops [17].

Since snowflakes form in clouds before traveling through the atmosphere and depositing on terrestrial surfaces, snow is tightly coupled to the atmosphere [18–20]. Therefore, the snowpack provides an opportunity to investigate the involvement of atmospheric microorganisms that have been linked to cloud development, atmospheric chemistry, and microbial biogeography [21, 22] in snow formation and their subsequent persistence in terrestrial snowpack communities. Microorganisms can serve as ice nucleation particles (INPs) that are required for snow crystal growth in clouds and are found in freshly fallen snow and other precipitation forms [23–26]. They are among the most active INPs [27], with bacteria such as *Pseudomonas syringae* able to initiate snow crystal growth at temperatures as high as −3°C [28]. Snow is an effective scavenger of atmospheric particles, including microorganisms. While previous research has suggested that snow might be used to investigate microbial communities in the atmosphere [29], their respective community structure and diversity are different [30]. Selective processes occur as snow falls in the atmosphere [17, 23]. Once on the ground, snowpacks at our study site are continually seeded by the atmospheric deposition of nutrients, contaminants, and other organisms that create a dynamic habitat that evolves over time [31–33].

Several theories developed in macroecology to explain local community structure, including niche-based theories [34] and neutral assembly theories [35], are now being applied to microbial communities (e.g., [36–38]). Niche-based theories state that deterministic responses to variation in environmental conditions and interspecific interactions drive community assembly [39], while neutral theories are based upon the functional equivalence of species [40] that implicates dispersal and stochasticity for community assembly during colonization and extinction events [41]. The current general consensus is that the structuring of microbial communities in a given environment is a result of both mechanisms [42, 43]. However, the relative contributions of neutral and niche-based mechanisms to community assembly in snowpacks have not been determined.

A variety of computational tools have been developed to study the structuring of microbial communities in environmental samples such as the neutral model approach developed by Harris et al. [44] based on Hubbell’s Unified Neutral Theory of Biodiversity (UNTB). In this model, a regional pool of trophically similar species, or metacommunity, is defined as the pool from which a local community, defined as a group of species that compete for resources in the local area, is hypothetically colonized [44]. The UNTB assumes that local communities are assembled/reassembled by the birth/death of individuals and immigration from the metacommunity and that deterministic competitive interactions between species are insignificant [45]. Immigration to local communities is assumed to be highest for abundant species in the metacommunity [44]; therefore, differences in environmental factors at the local scale should not influence community structure. The Harris model calculates this metacommunity from the taxonomy of all of the samples, an approach that is useful when the microbial seeding source is difficult to sample, but is tightly linked to the individual local communities. This may be the case for the examples used to test the method such as for microbiomes of tropical trees and the human gut [44]. The advantage of the seasonal snowpack ecosystem is that freshly fallen snow is the main seeding source, because it consists of both microorganisms in clouds (where snowflakes form) and those scavenged from the underlying air during snowfall [30]. The surface upon which the seasonal snow develops might also constitute a seeding source, in seasonal snow found on glaciers; this would either be ice or previously fallen snow or firn (aged snow older than a year). Once the glacier ice or firn surface is covered with the first snowfalls, it is spatially segregated from the over-lying snow layers; therefore, we consider that thereafter, the atmosphere is the main source of microorganisms. An important question is whether this snow constitutes a good proxy of the metacommunity or whether the above-defined metacommunity by the UNTB model is a good approximation of the seeding source.

In this study, the winter-spring snowpack was sampled in April 2016 across the Norwegian Arctic archipelago of Svalbard as part of the Community Coordinated Snow Study in Svalbard (C2S3). This survey mapped variations in the biological and chemical composition and impurity load of the snowpack across glaciers of Svalbard and related the observed differences to meteorological and other environmental factors [46, 47]. Here, we applied the UNTB model to snow from a range of different sites to evaluate the possible contribution of neutral assembly to the winter-spring snowpack microbial communities, and test whether this model is appropriate to predict them. Additionally, we assess possible niche-based effects by evaluating the effects of chemical and geographical variation on snow independent of the ecology model application. Specifically, the aims of this study were (1) to identify underlying patterns in community structure in seasonal glacial snowpacks across the scale of Svalbard, collected prior to the onset of melt, (2) to evaluate the extent to which niche based and neutral processes contributed to biodiversity, and (3) to identify the drivers for microbial selection in snowpacks.

## Methods

### Sampling location and strategy

**Fig. 1:**
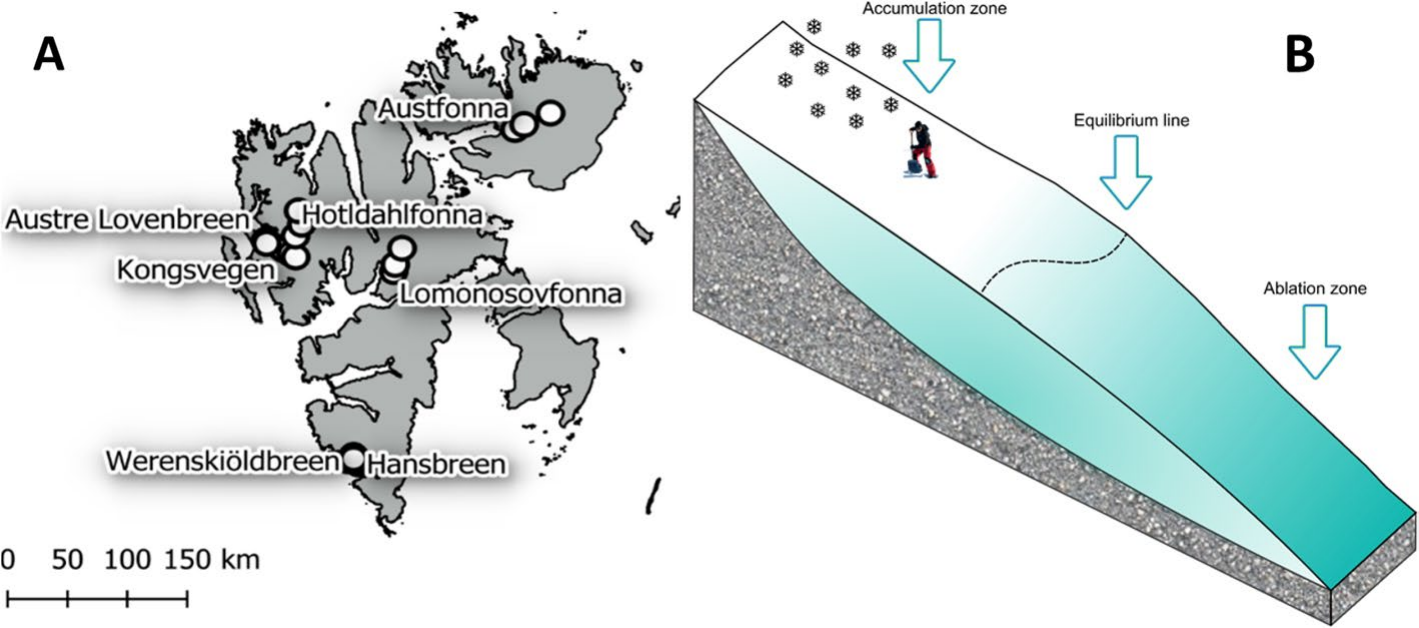
Map of glaciers and sites sampled, spanning 102–1193 m above sea level (a.s.l.)

The data presented here were obtained as part of a comprehensive survey of the physical, chemical, and microbiological properties of the seasonal snowpack carried out in April of 2016 at 22 sites distributed across seven glaciers on Spitsbergen and Nordauslandet, in the Norwegian Arctic Archipelago of Svalbard (Fig. 1, left panel). On each glacier, a total of 3 snowpits were sampled, one in the net accumulation area (highest elevations), one near the equilibrium line (where net annual snow accumulation equals summer melt, Fig. 1, right panel), and one in the net ablation area (lowest elevations) [48]. The sampled glaciers cover a wide range of elevations and geographic regions of the Svalbard Archipelago and share enough glaciological similarities for intercomparison: Austfonna on Nordaustlandet (Eastern Svalbard); Lomonosovfonna on central Spitsbergen; Hansbreen and Werenskiöldbreen in the Hornsund area of southern Spitsbergen; and Austre Lovénbreen, Kongsvegen, and Holtedahlfonna in the vicinity of Ny-Ålesund on northwestern Spitsbergen (Fig. 1, Table 1). Snowpits were sampled down to the hard previous summer surface (in the accumulation zone of glaciers), or to the underlying bare ice surface (in the ablation zone) using a common protocol [49] with pre-cleaned equipment (i.e., tubes, plastic scrapers, and plastic shovels cleaned with ultrapure water) and protective clothing (powder-free nitrile gloves, clean coverall suits, and face masks). Therefore, only the snow that accumulated over the past year (annual) was sampled. All snow pits were located away from point sources of contamination (e.g., field camps, snow scooter tracks) and were accessed by foot only in the upwind direction. After recording the snowpack stratigraphy, parallel samples were collected from the top 5 cm, and at 50-cm-depth intervals beneath for inorganic elemental carbon (EC) and WIOC (water-insoluble organic carbon) and microbial analyses. Snow samples were double-bagged in sterile LDPE bags (Whirlpak®) and packed in styrofoam boxes during transport. All samples were kept frozen until processed in the lab and further analyzed. A total of 89 samples were obtained from all sites. Samples for ionic chemistry (major and minor ions) and organic carboxylic acids were also collected from each discrete snow pit layer, according to the visible stratigraphy, and pooled to similar depths for comparison with microbiology and EC and WIOC. Organic acids were analyzed for all samples, with the exception of those collected in the Hornsund area. The chemical samples from Hornsund were analyzed on site and the instrument was not calibrated for organic acid analyses, whereas all other samples were analyzed in Venice (see below). Two companion papers that detail both the analytical procedures and results of the EC and WIOC loading and distribution [46] and ionic chemistry [47] of these glaciers have recently been published as part of this project.

**Table 1:** Location of each glacier sampled with corresponding estimated diversity and abundances of bacterial communities. Values present averages (bold) as well as ranges (in brackets). Letters show significance levels comparing diversity estimates by pairwise comparisons using the Wilcoxon rank-sum test

Glacier	GPS coordinates	Shannon index	Abundance	SWE-corrected abundance
			[16S rRNA gene copies L^**−**1^_melted snow_]	[16S rRNA gene copies m^**−**2^_snow_]
AF	79.7669° N	**1.68**	**1.5e+5**	**2.6e+6**
	22.8475° E	(1.33–3.35)^a^	(3.3e+4–1.2e+6)	(6.1e+4–2.2e+7)
ALB	78.8719° N	**3.17**	**8.4e+5**	**1.1e+7**
	12.15916° E	(1.29–4.20)^b^	(3.3e+4–6.7e+6)	(7.1e+4–9.3e+7)
HDF	79.02936° N	**2.92**	**5.0e+5**	**6.91e+6**
	13.53098° E	(1.25–3.89)^b^	(2.0e+4–4.3e+6)	(1.4e+4–6.0e+7)
KVG	78.78049° N	**3.61**	**4.3e+5**	**4.0e+6**
	13.15344° E	(1.65–4.34)^b^	(3.0e+4–3.6e+6)	(6.4e+4–1.9e+7)
LF	78.69132° N	**2.36**	**1.2e+5**	**1.0e+6**
	17.14985° E	(1.12–4.00)^b^	(4.2e+4–2.6e+5)	(1.5e+4–3.2e+6)

### Chemical analyses

Detailed methods and results for EC, WIOC, and ionic chemistry analyses of the C2S3 snow samples are reported in companion papers [45, 46]. Only a brief summary is provided here. The mass concentration of EC and WIOC filtered from snow was determined by the thermo-optical method at the Department of Environmental Science of Stockholm University [45]. Inorganic ion analyses were jointly performed by ion chromatography at the Polish Polar Station Hornsund (Institute of Geophysics, Polish Academy of Sciences), the Department of Earth Sciences at Uppsala University (Sweden), and at the Institute of Polar Sciences (ISP-CNR) in Venice [46]. The species measured were sodium (Na^2+^), ammonium (NH_4_^**+**^), calcium (Ca^2+^), magnesium (Mg^2+^), potassium (K^+^), bromide (Br^**−**^), chloride (Cl^**−**^), sulfate (SO_4_^**−**^), nitrate (NO_3_^**−**^), and methanesulfonic acid (MSA; as CH_3_SO_3_^**−**^) [46]. The organic acids were measured at ISP-CNR in Venice and included C1-formic, C2-oxalic, C2-acetic, C2-glycolic, C3-malonic, C4-succinic, C5-glutaric, and C6-adipic acids. Briefly, the chromatographic separation of these species was conducted using an anion exchange column (Dionex Ion Pac AS 11 2 × 250 mm) and a guard column (Dionex Ion Pac AG11 2 × 50 mm). The gradient of sodium hydroxide (NaOH), produced by an eluent generator (Dionex ICS 5000EG, Thermo Scientific), with a 0.25 mL min^−1^ flow rate was as follows: 0 min, 0.5 mM; 0–3.5 min gradient from 0.5 to 5 mM; 3.5–5 min gradient from 5 to 10 mM; 5–25 min gradient from 10 to 38 mM; 25–30 min, column cleaning with 38 mM; 30–35 min; equilibration at 0.5mM. The injection volume was 100 μL. A suppressor (ASRS 500, 2 mm, Thermo Scientific) removed NaOH before entering the (−)-ESI source of a single quadrupole mass spectrometer (MSQ Plus™, Thermo Scientific™) that operated in Single Ion Monitoring (SIM) mode [50].

Snow is a porous media [51], with density generally varying from 50 to 400 kg/m^3^ in pre-melt seasonal Arctic snowpacks. Therefore, to ensure comparability across snow samples of different densities, the measured concentrations of chemicals were expressed as mass loadings (mg m^**−**2^). The mass loading is calculated as the chemical concentration multiplied by the snow water equivalent (SWE, in mm of equivalent water) of each discrete layer where the SWE is calculated using the layer thickness and the density. This flux is representative of the mass accumulation for each studied layer/sample increment. The chemical and metadata for the whole data set used for comparisons are presented in SI Table 2.

### DNA extraction and 16S rRNA sequencing

Snow samples (*n*=89) were melted at room temperature at the laboratories at the University Centre in Svalbard (UNIS) and filtered immediately after melting. Procedural blanks (*n*=6) were carried out using the UNIS laboratory facility’s Milli-Q water system. All samples were filtered using sterile 0.2-μm filters (Merck Millipore) and a filtration unit connected to a vacuum pump. Filters were then stored at −20°C and shipped to the laboratory at the University of Lyon for further processing. DNA was extracted using the Power Water DNA isolation kit (Qiagen), following the manufacturer’s instructions. The V3–V4 region of the 16S rRNA genes from snow samples was amplified by a PCR of 35 cycles at 92 °C for 30 s, 55 °C for 30 s, and 72 °C for 60 s with the primer pair S-D-Bact-0341-b-S-17/S-D-Bact-0785-a-A-21 from [52], using the Platinum PCR SuperMix (Invitrogen™). Libraries for 16S rRNA sequencing were prepared using the “16S rRNA gene Library Preparation Workflow” recommended by Illumina. The purified (AMPure XP beads, Beckman Coulter) libraries of single samples were measured spectrophotometrically and subsequently pooled equimolar. This pool was checked for correct library construction and absence of primer dimers on the Bioanalyzer 2100 system (Agilent, DNA 1000 assay) and quantified by qPCR using primers annealing to the P5 and P7 flanking regions of the library. The final pool was loaded on a V2 flow cell for 2×250 bp paired-end sequencing on a MiSeq platform (Illumina) at the laboratory in Lyon. Evenly distributed base quality scores of forward and reverse reads throughout all samples were controlled using the functions fastx_quality_stats and fastq_quality_boxplot_graph of the FASTX-Toolkit (http://hannonlab.cshl.edu/fastx_toolkit/). PANDAseq [53] was used to merge forward and reverse read using the rdp_mle flag as merging algorithm. Each resulting sequence was stripped of its primers and annotated using the RDP Classifier [54] (cutoff = 0.6). Taxonomic strings only found one time were removed. Samples were blank corrected for the procedural blanks (*n*=6) using the decontam package in R [55]. Briefly, procedural blanks prepared during sample treatment were used to define a set of “negative control” samples. The frequency and prevalence methods were then used to identify potential contaminants and remove these from the snow samples.

### Quantitative PCR

Microbial abundance was estimated by quantitative PCR (qPCR) of the 16S rRNA gene, which was performed on a Corbett Rotor-Gene 6000 real-time PCR cycler and QuantiTect SYBR® Green PCR Kit (Qiagen). Amplicon quantitative PCR is often used to estimate total microbial abundance based on 16S rRNA gene copy number [56]. This approach is inherently biased, since there is over an order of magnitude 16S rRNA gene copy number variation in bacteria [57] and estimates should be considered as semi-quantitative and not absolute. Primer sequences used were 341F (CCTACGGGAGGCAGCAG) and 534R (ATTACCGCGGCTGCTGGCA) from [58]. Standard curves of all reactions were derived from serial dilutions of linearized pGEM-T plasmids (Promega) with the target sequences inserted. All standard curves were linear and showed comparable efficiency values (*R*^2^ = 0.96 to 0.99; *E* = 0.85 to 1.05). Each reaction (25 μL) contained 12.5 μL 2× QuantiTect SYBR® Green Mix; 0.3 to 1.8 μL of each primer (10 μM); 100 ng of T4 gene protein 32 (Thermo Fisher Scientific Inc.); 2 to 5 ng of template DNA and PCR grade water. Two-step cycling conditions were 3 min at 95°C followed by 30 cycles of 5 s at 95°C and 30 s at 60°C. Results from runs with no amplification in non-template controls (*n*=3 per run), showing only one melting peak, and overlapping peaks from standards were considered for further analysis.

### Neutral community modeling and identification of potential ice nucleation active (pINA) organisms

Using a Bayesian fitting strategy to evaluate Hubbell’s UNTB at multiple sites, we tested for neutrality and defined immigration rates at different taxonomic levels using the software described in Harris et al. [44]. The model was only fit to a taxon (with at least 150 representatives) if it was detected in at least 50 samples. Individual samples (local communities) from each snow layer and each glacier (*n*=89) were assessed for their fit to a neutral model and all samples taken together were defined as the metacommunity and analyzed. The neutral assembly model parameters were estimated by modeling the data as a hierarchical Dirichlet process (HDP, [44]). Genera that were potentially INA were identified in each sample by blasting (blast version 2.7.1) against a nucleotide database of organisms containing the ice nucleation protein gene as outlined in [59], with a threshold of 97% similarity. Briefly, the 16S rRNA gene sequences of total sequenced bacterial genomes containing genes that encode ice-nucleating proteins were downloaded from the NCBI database and used to identify potential ice nucleators. This approach can only be used to identify potential ice nucleators, since carrying the ice-nucleating genes does not equate ice nucleation activity. The percentage of potential ice nucleators per bacterial class was calculated for the entire dataset.

### Community analysis

All community analysis was carried out using Phyloseq package [60] in R [61]. Diversity indices were calculated for each sample to describe richness and evenness (SI Table 3). Cell counts were corrected for snow density prior to analysis (as described for chemistry). PERMANOVA on a Bray-Curtis dissimilarity matrix of sample counts was performed using the *adonis* function of the vegan-package in R (1000 iterations) to test for significant differences between sample clusters, sampling zones, and sampling layers [62]. Pairwise comparisons and the Wilcoxon rank-sum test were carried out to compare diversity, zone, glacier, layer, and clusters. These analyses were applied to the subset of data for which organic chemical data was available (59 samples from all glaciers with the exception of those from the Hornsund area). Clustering of samples based on community structure was carried out in Phyloseq using the gap statistic and validated using hclust on the subsampled data (SI Figure 1). Adonis and Deseq2 were used to identify significantly different genera among sample clusters in R and pairwise tests were also used to compare diversity.

### Variance partitioning analysis

Variance partitioning analysis (VPA) was used to test for significance of chemical and geographical variables in determining community structure (*n*=59). All statistical analyses were performed using the statistical software R with packages VEGAN [62] and Phyloseq [60]. Redundancy analysis (RDA) was performed on *Z*-transformed log-transformed chemical data and Hellinger transformed microbiological data as described by [63]. Geographical data, including site coordinates, snow layer, and sampling zone (ablation, equilibrium, and accumulation) were also used as explanatory variables. Forward selection was carried out using the ordistep function in VEGAN for variable reduction [63]. Variation partitioning [64, 65] was used to test the significance of the contribution of geographical and chemical data.

### Multivariate analysis, correlations, and predictive modeling

Different types of statistical analyzes, as outlined below, were carried out in R to link the microbial data to the chemical data set using the ade4 package [66]. Co-inertia (CIA) [67] was carried out on the combined log-transformed chemical and microbial data, following Z- and Hellinger transformation respectively [68], to study the relationships between chemistry (inorganic and organic chemistry) and microbial community structure as well as spatial and temporal variations simultaneously [69]. The initial analysis was carried out on the entire data set for which both chemical and microbiological data was available (*n*=89); however, no significant co-structures were observed. The analyses were subsequently carried out on the subset of data (excluding the Hornsund area) for which organic chemistry was also available (*n*=59). A randomization test of 1000 permutations was carried out to verify the significance of the co-structure (Monte Carlo test) [70]. Spearman correlations were calculated to test correlations in the whole data set using the Hmisc R package with *p*-values adjusted using the false discovery method [71].

## Results

### Neutral modeling

When applying the UNTB model from Harris et al. [44] to test for neutral community assembly, both the metacommunity and the local community at the genera level did not meet the criteria for neutral assembly (pseudo-*p* = 0 and 0.0004, respectively; Table 2). This was also the case for local communities at the class and family level (pseudo-*p* = 0.0012 and 0.0004, respectively). However, when subsets of the taxa data from a single class were considered (e.g., genera in *Actinobacteria*, *Bacilli*, *Betaproteobacteria*, *Cyanobacteria*, *Cytophagia*, *Gammaproteobacteria*), neutral assembly processes were likely at both the metacommunity and local levels in all cases (pseudo-*p* > 0.2) except for *Gammaproteobacteria* that did not meet the criteria for neutral assembly at the metacommunity level (pseudo-*p* = 0.0024). *Pseudomonas*, the most dominant genus in the *Gammaproteobacteria*, appeared to be neutrally assembled at both the metacommunity and local community levels.

**Table 2:** HDP modeling outputs to test for neutrality in the meta and local communities (*n* = 89). Tests were carried out at different taxonomic resolutions. Theta: rate at which new individuals appear in the metacommunity through speciation; immigration: calculated by the HDP model; *pN*: probability of neutrality in the metacommunity; *pL*: probability of neutrality in the local community; *lc*: lower quartile; *med*: median; *uc*: upper quartile

	Theta			Immigration			pN meta community	pL local community
	lc	med	uc	lc	med	uc		
**Taxonomic level**								
Phylum	2.5	3.9	5.7	4.7	10.7	21.9	0.49	0.0784
Class	9.2	11.9	15.1	9.4	15.8	25.5	0.38	0.0012
Family	47.7	54.4	62.1	20.5	28.3	38.5	0.21	0.0004
Genera	158.5	172.5	187.7	28.9	37.8	48.9	0.0000	0.0004
**Class**								
*Actinobacteria*	27.4	32.6	38.4	13.3	21.6	34.2	0.16	0.6
*Bacilli*	14.9	19.0	23.9	3.7	7.7	14.9	0.16	0.8
*Betaproteobacteria*	14.1	17.9	22.5	5.1	10.4	20.3	0.20	0.8
*Chloroplast*	0.2	0.5	1.4	0.4	2.6	11.8	0.86	1.0
*Cyanobacteria*	0.3	0.8	1.8	0.4	4.0	25.6	0.50	1.0
*Cytophagia*	2.7	4.3	6.5	1.9	5.8	16.3	0.20	0.9
*Gammaproteobacteria*	16.4	20.5	25.5	3.9	7.9	14.9	0.0024	0.3
*Genus: Pseudomonas*	0.5	1.1	2.1	0.6	6.2	45.6	0.31	1.0

### pINA bacteria

To estimate the contribution of INA to the snowpack microbial community structure, we compared the reads of pINA bacteria to the total reads in each sample (List available in SI). The highest amount of pINA bacteria was related to *Gammaproteobacteria*, which comprised 25% of the overall reads, followed by *Bacilli* (5%) and *Flavobacteria* (1%). Within the *Gammaproteobacteria*, pINA genera represented almost 75% of the reads and were dominated by *Pseudomonas* (Table 3). When comparing abundance-corrected values for pINA bacteria (SI Table 1), the quantity did not change significantly from sample to sample, yet the relative abundance did. In other words, absolute numbers of pINA bacteria remained fairly constant even though many samples had an overall increase in bacterial numbers. There was no correlation between relative abundance and estimated amount of pINAs across the dataset (SI Figure 2).

**Table 3:** Abundance of potential ice nucleators (pIN) in the various bacterial classes identified based on the total sequenced 4,177,068 reads

Class	pIN reads	pIN in dataset [%]	Total reads in Class	pIN in Class [%]
*Actinobacteria*	570	0.01	605,648	0.09
*Alphaproteobacteria*	1696	0.04	16,963	10
*Bacilli*	215,598	5.16	632,001	34.11
*Betaproteobacteria*	6213	0.15	239,389	2.6
*Clostridia*	186	0	17,608	1.06
*Cyanobacteria*	0	0	0	0
*Deinococci*	240	0.01	9732	2.47
*Deltaproteobacteria*	2	0	36,900	0.01
*Flavobacteriia*	42,496	1.02	46,955	90.5
*Gammaproteobacteria*	1,056,951	25.3	1,420,310	74.42
*Planctomycetia*	1220	0.03	16,304	7.48

### Community structure, diversity, and bacterial abundance

**Fig. 2:**
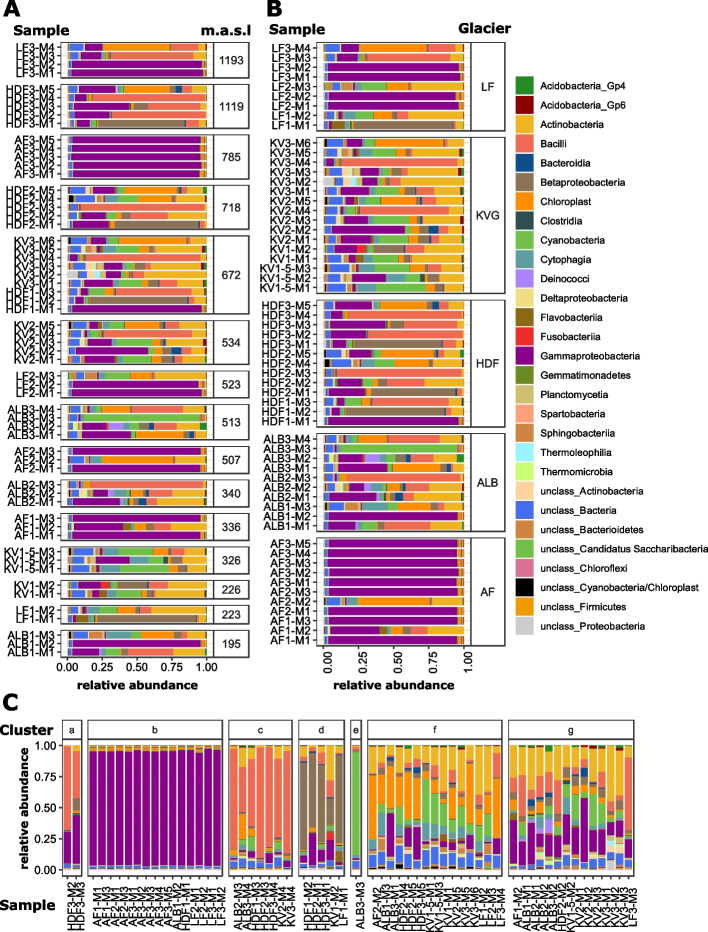
Relative abundances of dominant classes from bacterial communities analyzed in single samples grouped by altitudinal height (m a.s.l = meters above sea level) (**A**), glacier (**B**), and cluster analysis (**C**). Samples are named as follows: Glacier abbreviation (AF=Austfonna, LF=Lomosofonna, HDF=Holtedahlfonna, KV= Kongsvegen, ALB= Austre Lovénbreen), followed by 1, 2, and 3, which represent either the ablation, equilibrium, or accumulation zone of the glaciers, respectively. The numbers following the M represent the individual snow layers, with M1 representing the top 5 cm of the snowpack and M2, M3, etc. representing the underlying layers in increments of 50 cm

No clear sample grouping based on glacier sampled or snow layer was apparent, although both were shown to have a significant correlation with community structure. Instead, samples were clustered into seven clusters based on their community structure (Fig. 2 panel C, SI Table 2): cluster a was dominated by *Bacilli* (51%), *Gammaproteobacteria* (23%), and *Betaproteobacteria* (5%). Cluster b was dominated by *Gammaproteobacteria*, represented on average over 75% of the relative abundance of taxa, and mainly clustered samples collected in Austfonna and Lomonosovfonna. A total of 53 genera were found to be significantly more abundant in cluster b relative to the rest of the data set (SI Table 4). Furthermore, cluster b had a significantly lower Shannon diversity index (see Table SI 3 for all richness estimates) as compared to all other clusters and community structure was also significantly different (SI Table 5). Cluster c was dominated by *Bacilli* (33%), *Chloroplast* (17%), *Gammaproteobacteria* (7%), and *Cyanobacteria* (6%) and represented samples collected in the mid to basal layers of Austre Lovénbreen, Holtedahlfonna, and Kongsvegen (Fig. 2). All 20 genera that were significantly more abundant in this cluster belonged to the *Firmicutes* phylum (SI Table 4). Cluster d, consisting of samples from Holtedahlfonna, Lomonosovfonna, and Kongsvegen, was dominated by *Betaproteobacteria* (53%, Fig. 2) and 3 significantly more abundant genera belonged to this class: *Janthinobacterium*, *Massilia* and unclassified *Oxalobacteraceae* (SI Table 4). Cluster e consisted of a single sample collected from Austre Lovénbreen that was characterized by a high relative abundance of *Cyanobacteria* (68%). Cluster f was characterized by *Chloroplast* (45%), *Actinobacteria* (27%), and *Cyanobacteria* (17%). A total of 44 significantly more abundant genera were found, representing *Actinobacteria*, *Sphingobacteria*, *Alphabacteria*, and *Chloroplasts*, among others (SI Table 4). Cluster g was dominated by reads associated to *Actinobacteria* (31%) followed by *Bacilli* (29%), *Cyanobacteria* (21%), *Gammaproteobacteria* (19%), and *Deinococci* (10%). Genera from the *Lactobacillales*, *Moraxellaceae*, and *Actinomycetales* orders represented the 11 significantly more abundant genera in this cluster. SIMPER analysis identified taxa with the most significant impact on overall community structure (SI Table 4), these included taxa such as *Pseudomonas*, *Acinetobacter*, *Shigella* and *Rhodanobacter* (*Gammaproteobacteria*), *Polaromonas* and *Oxalobacteraceae* (*Betaproteobacteria*), *Chloroplasts* and *Cyanobacteria*, *Actinobacteria* and *Bacilli*.

Differences in community structure (PERMANOVA using Bray-Curtis dissimilarity matrix) and diversity (Shannon index, Wilcoxon rank-sum test, Holm adjustment method) among zone (ablation, equilibrium and accumulation), layers (surface, middle, basal), and glaciers were also explored (SI Table 5). No significant differences in community structure and diversity were observed among zones, while significant differences in community structure were only found between surface and basal samples (*r*^2^ = 0.07, *p*=0.04). Significant differences in community structure and diversity were found between Austfonna and Austre Lovénbreen (*r*^2^ = 0.26, *p* = 0.01 and *p* = 0.026, respectively), Austfonna and Holtedahlfonna (*r*^2^ = 0.23, *p* = 0.01 and *p* = 0.025, respectively), Austfonna and Kongsvegen (*r*^2^ = 0.33, *p* = 0.01 and *p* = 1.8e−05, respectively), and Kongsvegen and Lomonosovfonna (*r*^2^ = 0.13, *p* = 0.03 and *p* = 0.026, respectively). Diversity was also significantly different between Holtedahlfonna and Kongsvegen (*p* = 0.023). Diversity was lower in Austfonna and Lomonosovfonna, and no differences were observed among the other glaciers. There was no significant effect of altitude on diversity. Changes in microbial abundance were not related to glaciers sampled or altitude, but we did see a significant effect of sample depth (*p* < 0.001), with surface samples having on average 1–2 log10-fold copy numbers of 16S rRNA genes less than middle and basal layers. There was a significant relationship between diversity, represented by the Shannon Index, and abundance (Table 5, *r*^2^=0.4, *p*=0.002).

### Variable partitioning analysis (VPA)

To determine the relative contribution between niche-based processes, VPA was performed. Following variable inflation analysis (VIF), five chemical variables (acetic acid, glutaric acid, malonic acid, EC, and NH_4_^+^) and three geographical variables (longitude, elevation, and snow layer depth) were selected for variation partitioning. Geochemical variables explained a total variance of 31.5% (*p* = 0.001), with chemistry accounting for 12.7% (*p* = 0.001), geography for 4.1% (*p* = 0.001), and a shared contribution of 14.7% (*p* = 0.003) (Table 4). The residual variance was 68.4.

**Table 4:** Variable partitioning analysis (VPA) to determine relative contribution to niche-based and neutral processes. Five chemical variables (acetic acid, glutaric acid, malonic acid, EC, and NH_4_^+^) and 3 geographical variables (gradient from east to west, elevation, and snow layer) were selected from the output of a variable inflation analysis (VIF)

Variable group	% variance in community structure explained	*p*-value
Chemistry	12.7	0.001
Geography	4.1	0.001
Chemistry + geography	14.7	0.003
Residuals	68.4	0.001

### Taxonomic links to chemistry

Based on multivariate analysis, we were unable to find a clear correlation between microbial taxonomy and snowpack chemistry when taking into account only the inorganic parameters measured (measured on all samples). When subsampling the data to include only samples for which organic chemistry data was available (*n*=59), a significant co-structure was observed using co-inertia analysis (CIA) (*p* = 0.001, RV = 0.35). The RV-coefficient represents the correlation between both datasets and varies between 0 and 1; the closer the coefficient is to 1, the stronger the correlation between the datasets [69]. The chemical parameters and their associated genera formed five major axes: (1) acetic and malonic acid, associated genera included *Solirubrobacter*, *Anabaena*, unclassified *Bacilli*, *Oxalophagus*; (2) NO_3_^**−**^ and WIOC deposited, associated taxa included *Bacilli* (e.g., *Ammoniphilus*, *Allobacillus*, *Bacillus*) and Actinobacteria (*Iamia*, *Dactylosporangium*, *Virgisporangium*); (3) NH_4_^**+**^ and Br^**−**^, associated taxa included *Nitrososphaera*, *Fictibacillus*, *Methylophaga*, *Marinobacterium*, and *Acholeplasma*; (4) methane sulfonic acid (MSA) and glutaric acid, associated taxa included *Escherichia/Shigella*, *Enhydrobacter*, *unclassified Porphyromonadaceae*, *Aerococcus*, *Brachybacterium*, *Burkholderia*, and *Acinetobacter*; and (5) EC deposited, associated with taxa *Azospira* and *Parachlamydia*. Several taxa corresponding to cluster b were negatively correlated to organic acid loading, including *Pseudomonas*, *Desulfomicrobium*, *Pelobacter*, *Desulfocurvus*, and *Glaciecola*, *Neptunomonas*, and *Oceanospirillaceae*.

### Linking abundance, diversity, and immigration to geochemical data

Correlation analysis was carried out to determine the main drivers of bacterial abundance, diversity, and immigration (Table 5, SI Figure 3). Bacterial abundance was positively correlated to Cl^**−**^ (*r*^2^ = 0.63), Na^+^ (*r*^2^ = 0.51), Ca^2+^ (*r*^2^ = 0.60), K^+^ (*r*^2^ = 0.26), EC and WIOC (*r*^2^ = 0.27 and *r*^2^ = 0.85, respectively), acetic, formic, malonic, and oxalic and succinic acid (*r*^2^ = 0.5, *r*^2^ = 0.51, *r*^2^ = 0.45, *r*^2^ = 0.46, *r*^*2*^ = 0.26, respectively), NO_3_^**−**^ (*r*^2^ = 0.59), and snow temperature (*r*^2^ = 0.36). Diversity, represented by the Shannon index, was positively correlated with abundance (*r*^2^ = 0.40), MSA (*r*^2^ = 0.45), organic acids: acetic (*r*^2^ = 0.57), formic (*r*^2^ = 0.58), glutaric (*r*^2^ = 0.35), glycolic (*r*^2^ = 0.28), succinic (*r*^2^ = 0.46), and malonic acid (*r*^2^ = 0.46). Immigration was correlated to acetic (*r*^2^ = 0.46), formic (*r*^2^ = 0.48), oxalic (*r*^2^ = 0.45), succinic (*r*^2^ = 0.45), malonic acid (*r*^2^ = 0.33) and to abundance (*r*^2^ = 0.41). Both the Shannon index and immigration were negatively correlated to the percentage of pINA bacteria (*r*^2^ = -0.83, *r*^2^ = −0.65, respectively).

**Table 5:** Correlations of measured parameters with diversity (Shannon), immigration and abundance (*n* = 59). Values in bold are significant at *p* < 0.05

	Shannon		Immigration		Abundance	
	corr.	*p*-value	corr.	*p*-value	corr.	*p*-value
Abundance	**0.400**	0.002	**0.411**	0.001	-	-
Acetic acid	0.572*	<0.001	**0.464**	<0.001	**0.503**	<0.001
Adipic acid	0.235	0.073	0.192	0.145	0.178	0.180
Br^**−**^	**−0.313**	0.016	**−**0.206	0.117	0.247	0.062
Ca^2+^	0.144	0.274	0.136	0.304	**0.602**	<0.001
Cl^**−**^	0.147	0.266	0.083	0.532	**0.628**	<0.001
EC	0.076	0.568	**−**0.065	0.627	**0.268**	0.041
Formic acid	0.580*	<0.001	0.474*	<0.001	**0.512**	<0.001
Glutaric acid	**0.349**	0.006	0.069	0.601	**−**0.117	0.381
Glycolic acid	**0.276**	0.034	0.147	0.267	**0.310**	0.018
Potential IN	−0.832*	<0.001	−0.649*	<0.001	**−0.472**	<0.001
K^+^	**−**0.235	0.073	**−**0.177	0.179	**0.263**	0.046
Malonic acid	**0.463**	<0.001	**0.329**	0.01	**0.448**	<0.001
Mg^2+^	0.008	0.949	**−**0.010	0.938	**0.373**	0.004
MSA	**0.447**	<0.001	0.209	0.111	**−**0.058	0.661
Na^+^	0.070	0.595	0.004	0.976	**0.514**	<0.001
NH_4_^+^	**−**0.235	0.074	**−0.267**	0.041	0.224	0.091
NO_3_^**−**^	**0.267**	0.041	0.227	0.084	**0.585**	<0.001
WIOC	**0.262**	0.045	0.175	0.185	0.847*	<0.001
Oxalic acid	**0.462**	<0.001	**0.453**	<0.001	**0.459**	<0.001
SO_4_^2**−**^	0.064	0.630	0.041	0.759	0.535*	<0.001
Succinic acid	**0.456**	<0.001	**0.341**	0.008	**0.262**	0.046
Temperature	0.203	0.112	0.207	0.116	**0.364**	0.005
Immigration	**0.825**	<0.001	-	-	**0.411**	0.001
Shannon	-	-	**0.825**	<0.001	**0.400**	0.002

## Discussion

Snow supports a high diversity of organisms, whose abundance and composition vary spatially and temporally [32, 72–78]. In our study on seven glaciers in Svalbard (Fig. 1), *Gammaproteobacteria* was the most dominant class of organisms and was found in all 89 samples. Other classes of organisms, such as *Betaproteobacteria*, *Alphaproteobacteria*, *Actinobacteria*, *Bacilli*, *Sphingomonas*, *Flavobacteria*, and *Cyanobacteria*, were present in most samples. This is consistent with observations from many different snow studies [5, 15, 33, 79, 80]. In our samples, microbial diversity, as calculated by the Shannon index, ranged from to 1.12 to 4.34, and cell counts, estimated by quantifying the amount of 16S rRNA gene copy numbers in each sample, ranged from 10^3^ to 10^6^ copies/L of melted snow. Although these values are similar to those previously reported [32, 72, 81, 82], their broad range reflects the heterogeneity of communities sampled in this study. In general, abundance and diversity were higher in snow samples collected at depth relative to the surface of snowpacks. The relative abundance of *Gammaproteobacteria*, which was highest in samples found in cluster b (Fig. 2C), decreased in samples with high bacterial abundance and diversity. Cluster b samples were found mostly in the surface layers of snowpacks, had the lowest estimated abundance, and likely most closely represented the seeding community, which we posit is the atmosphere, since they were dominated by pINA-associated bacteria. The possible processes responsible for the differences in microbial communities (Fig. 2) could be associated with random sampling (neutral assembly) of the seeding source (pool of organisms found in clouds, snow, rain, and aerosols), post-depositional selection (niche-based assembly), or a combination of the two. Here, we applied several approaches to answer this question based upon the two proposed assembly theories and multivariate analyses.

### Neutral processes in structuring snowpack microbial communities

To address whether neutral assembly processes were significant in snowpack communities, we applied the neutral model approach developed by Harris et al. [44]. In this model, the microbial communities in samples collected at different snow depths and glaciers across Svalbard constitute the local communities. Local communities are assembled from the metacommunity, defined as the average of the local microbial communities by the model. The metacommunity is itself considered to be an approximation of the unknown seeding community. Using the Harris et al. [44] approach [44], neutrality at both the local and metacommunity was tested using hierarchical Bayesian modeling. We found that at class level resolution and above, neutrality was generally observed at the metacommunity scale. However, at finer taxonomic resolutions (genera), neutrality was not observed, suggesting environmental factors at the local scale influence community structure. At the class level, neutrality was observed for the metacommunity (*p* = 0.4), while at the local community level, niche-based processes dominated (*p* = 0.0012). Given that when one subset of a taxon is non-neutral, the whole guild appears non-neutral, we subsampled the data to the dominant classes and re-ran the analysis. Only *Gammaproteobacteria* exhibited non-neutral behavior at the metacommunity level, suggesting active selection, possibly due to its improved dispersal ability related to ice nucleation processes [83]. Conversely, *Gammaproteobacteria* exhibited neutrality at the local scale, suggesting the taxa found within this class share the same potential to colonize individual snow samples. As mentioned previously, cluster b in Fig. 2C is the most similar to the seeding source, yet it had the smallest immigration rates from the metacommunity. Communities from samples that had undergone adaptation to the snowpack environment had the largest immigration values. Therefore, the Harris approach calculated a metacommunity that was distant from the actual seeding source community (which conceptually could be considered the metacommunity, also).

### Importance of ice nucleation

Snowflake formation requires the presence of ice-nucleating particles, such as dust, pollen, or other aerosols. Recently, biological ice nucleation has garnered scientific attention, with ice nucleation active (INA) bacteria being among the few INPs that are active between −10 and 0°C, and therefore have the potential to be key ice nucleators under warmer temperatures in mixed-phase clouds [84]. In our study, *Gammaproteobacteria* were the most dominant class of bacteria and were found in all 89 samples. Specifically, *Pseudomonas*, which represented up to 90% of the taxa in some samples, are known ice nucleators, having been previously observed in snow and cloud water [25, 85–89]. We hypothesize that the active selection of *Gammaproteobacteria* into the metacommunity and the seeding community is in part due to the large proportion of ice nucleators in this class. Once formed, snow transits through the atmosphere and colonization likely occurs stochastically, depending on atmospheric residence time, the specific surface area of the snowflakes, relative humidity, and the sources of the underlying air masses. As such, we suggest that the neutral behavior exhibited by all other classes might be due to their random incorporation during snowfall.

Although ice nucleation potential may explain the selection of *Gammaproteobacteria* in the seeding community (falling snow), the capacity to nucleate does not guarantee post-depositional success. Based on our results, the relative abundance of potential ice nucleators decreased in the active snowpack. Only one site (Austfonna) appeared to lack a significant natural snowpack community development and had a relatively elevated proportion of (putative INAs) *Pseudomonas*. In all cases, *Gammaproteobacteria* estimated abundance did not decrease significantly with depth. On the other hand, the abundance of other genera in the samples increased either through the growth/selection of non-*Gammaproteobacteria* organisms or increased immigration into the sample if physically possible. Immigration is a key stochastic process in the UNTB [44] and based in part on the similarity of a local community to the metacommunity (roughly similar to the average of the local communities). A local community closely resembling the metacommunity would, therefore, have an apparent high immigration rate. The proportion of ice nucleators was negatively correlated to the calculated immigration rates, further supporting our hypothesis that they are indicative of the seeding source but not of the average snowpack community (model calculated metacommunity).

### Impact of geography and chemistry on microbial community structure, diversity, and abundance

To evaluate the importance of niche-based selection on snow communities and identify possible drivers, we used VPA to correlate chemical and geographical variables with microbial community structure. Both sets of parameters were shown to correlate significantly with snowpack microbial community structure, and together explained slightly over 30% of the total community variance. The residual variance may be explained by neutral assembly processes or variables not measured in our study—such as irradiance and other organic/inorganic compounds [2, 10, 31, 90].

Significant geographical drivers of snow community structure in our dataset included longitude, elevation, and sample depth within the snowpack. The glaciers included in the VPA generally exhibited an east to west increase in community diversity as estimated by the Shannon index (Table 1). This gradient in diversity might be due, in part, to the influence of the West Spitsbergen Current (WSC), a branch of the Atlantic Meridional Overturning Circulation that flows northwards along western Spitsbergen, bringing milder temperatures and higher precipitation to this sector of Svalbard compared to the central and eastern parts [91]. Based on deuterium excess values, Barbaro et al. [47] found that snow on Austfonna is likely partly precipitated from more northerly moisture sources than that deposited elsewhere in the archipelago. This different seeding source and hypothetical metacommunity would need to be treated separately.

The influence of geographical features on snow composition is further supported by the detection of specific taxa. For example, samples collected from glaciers geographically close to areas with first-year sea-ice cover (FYI; Austfonna and Lomonosovfonna) [47], were mostly in cluster b. The genera that were significantly more abundant in this cluster compared to the remainder of the dataset included archetypical sea-ice organisms such as *Glacieocola* [92], *Neptunomonas* [93], *Oceanospirillaceae*, found in snow over sea-ice [17], and *Rhizobiales*, identified in frost flowers [94]. Anaerobic microorganisms involved in sulfur cycling and mercury methylation were also significantly more abundant in cluster b, such as members of the *Deltaproteobacteria* (including *Desulfovibrio*) recently identified in winter sea-ice [95–97], further supporting the hypothesis of a close sea-ice source.

Changes in community structure were also shown to depend on elevation and snowpack sampling depth. Samples collected in the accumulation area of Holtedahlfonna and Lomonosovfonna (cluster a), both located >1000 m a.s.l., showed a high relative abundance (up to 77%) in *Bacilli*, especially in the lower snow layers that correspond to winter accumulation. The dominant genera in these samples included *Paenibacillus* and *Domibacillus*, both identified as potential ice nucleators, as well as *Bacillus*. These have commonly been found in free troposphere samples [98–102] and are hypothesized to survive by traveling as environmentally resistant endospores. This is supported by Barbaro et al. [47] who found that the glacier snowpit sites above 600–700 m a.s.l. were proportionally more influenced by ions from long-range transport and less by ions emitted from proximal marine sources.

The ionic composition of snow deposited on Svalbard glacier sites below ~600 m a.s.l. is known to be strongly affected by local sea-spray emissions [46, 100, 101]. Therefore, we hypothesized that events that can alter marine microbial composition, such as algal blooms, should be detected in snow samples. MSA is a commonly used marker of marine primary production [103], because it is a photo-oxidation product of dimethylsulfide (DMS) released in the waning stages of phytoplanktonic blooms [104, 105]. In our samples, MSA correlated to glutaric acid, with the highest concentrations generally found in surface snow (cluster d and g in Fig. 2C) or basal snowpack samples of low altitude glaciers. The samples at the base of the snowpack were likely influenced by the late season, i.e., autumnal algal blooms may occur. Surface samples with high MSA and glutaric acid concentrations likely reflected springtime bloom events that generally occur in April–May in Svalbard [106, 107]. Algal blooms are associated with dynamic shifts in microbial communities during different stages, with *Flavobacteriia* dominating during the peak, stationary, and decline phases [108–110]. *Betaproteobacteria* appear in the waning stages of blooms [110] and colonize the sinking particles of algal detritus [111]. The community structure in cluster d was dominated by *Betaproteobacteria*, specifically *Oxalobacteraceae*, such as *Janthinobacterium* and *Massilia*, that are able to break down complex organic matter [112]. *Janthinobacterium* are rarely detected in oceans, but blooms have been shown to occur in polar waters [113] and were recently observed to dominate May post bloom air samples in Svalbard [22]. The *Flavobacterium* genus was also significantly more abundant in cluster d relative to other samples and is reported to catalyze the degradation of algal polysaccharides [114, 115]. Genera such as *Acinetobacter*, *Shigella*, *Brachybacterium*, and *Bulkholderia* were significantly correlated to MSA and all significantly more abundant in cluster g. Members of the *Burkholderia* and *Acinetobacter* genera have been shown to degrade dimethylsulfoniopropionate (DMSP) [116], while species of the *Shigella* genera have been shown experimentally to produce DMS [117].

### Potential seeding sources

Inorganic ion loading of Cl^**−**^, Na^+^, abundant in sea spray, were significantly correlated to estimated bacterial abundance. This would suggest that marine environments constitute a bacterial colonization source for snowpacks. Correlations with microbial abundance can provide information on alternative sources, for example, 16S rRNA gene abundance was linked to Br^**−**^ and K^+^ concentrations, suggesting a sea-ice and terrestrial source, respectively, and the strong correlation with WIOC (*r*^2^ = 0.85) suggests co-transport or binding of bacterial cells to organic-rich particles. Several snow samples were dominated by genera corresponding to halotolerant freshwater *Cyanobacteria* (e.g., *Anabaena*) and *Chloroplast* sequences from snow algae, *Chlorophyta* (sample clusters e and f). These samples were mainly collected in the bottom layers of snowpacks that were closest to the glacier firn or ice surface in the equilibrium and accumulation zones. The basal snow samples corresponded to winter snowfall events (December–January) [46]. The autotrophs might have been redistributed to the base of the snowpack due to meltwater percolation during surface thaws (or rain-on-snow events) as is the case for EC and WIOC and other impurities [46, 118, 119]. However, no measured chemical parameters, including EC and WIOC, were linearly correlated to autotroph relative abundance. Alternatively, snow algae might overwinter on glacier surfaces prior to recolonizing the snow [120, 121]. However, the increased abundance in autotrophs in these layers might be a result of their activity within specialized niches, although activity measurements were outside the scope of this study.

### Post-depositional selection and potential activity

Previous time series studies on the evolution of microbial communities in Arctic terrestrial snowpacks suggest that post-depositional selection can occur, and this is linked to changes in the chemical environment [31–33, 87]. Recently, organic acids were shown to drive microbial activity and interactions in terrestrial snowpacks [10, 16]. When attempting to correlate community structure with chemistry in this study, the only significant parameters were organic acids. Organic acids, specifically acetic acid, were correlated to diversity (Shannon index), and VIF analysis showed that both acetic and malonic acid were the best predictors of community structure. Apparent immigration (as calculated by the neutral theory model), diversity, and abundance were also all significantly correlated with organic acids, suggesting that niches with higher organic carbon loadings might be hotspots for bacterial activity and growth. This growth would then be included in the model calculation of the metacommunity and possibly prevent the model from accurately estimating immigration rates. *Bacilli* (dominant in samples from cluster c) were significantly correlated with acetic, oxalic, and malonic acids, NO_3_^**−**^**,** and deposited WIOC concentrations in snowpacks. *Bacilli* are versatile N-cyclers [122] and some strains can produce mixtures of lactic, isovaleric, isobutyric, and acetic acid [123], while other organic acids, such as glycolic, oxalic, malonic, and succinic acid are produced by phosphate-solubilizing bacteria and fungi [124–126]. Laboratory studies conducted at −5°C in the dark using snow from Svalbard recently showed that heterotrophic communities were able to metabolize dissolved inorganic nitrogen, leading to increased abundance and the selection towards N-metabolizing genera such as *Bacillus* and *Caulobacter* [11]. In our data set, increases in estimated cell abundance and diversity were also correlated to inorganic nitrogen species (Table 5). Cell abundance was also positively correlated with snow temperature. Snow samples with higher density had higher numbers of ice lenses and ice layers, suggesting the potential presence of anaerobic microhabitats. Given that *Bacilli* are facultative anaerobes that can potentially switch to N-oxide respiration when O_2_ becomes limited, they might have an advantage in anaerobic habitats [122]. While activity was not measured in our study, these results highlight the need for targeted laboratory studies to quantify the role of microorganisms in organic acid metabolism and their potential growth in snowpacks.

## Conclusions

Our results show that deterministic processes structure local snow microbial communities when the snow community is active, while stochastic processes affect the microbial communities that arrive via the fresh snow with the exception of ice nucleators. Initial seeding was shown to be facilitated by the capacity to nucleate ice, although this did not ensure post-depositional success, since potential ice-nucleating capacity was negatively correlated to immigration. Once in the snowpack, the microbial community evolved in part due to the availability of organic substrates. Sites and samples that did not receive significant amounts of carboxylic organic acids remained similar to the seeding community. Organic acids were the most significant predictors of microbial diversity. While inorganic chemistry was not linked to diversity, it can be used to help identify dominant colonization sources and to predict microbial abundance, which was tightly linked to sea air. The general assumption of the UNTB is that the metacommunity, calculated from the taxa in the local communities, represents the seeding community. However, in our snow samples, this was not the case. The similarity of samples with high organic acid concentrations to mature snowpack microbial communities drove the generalized snowpack microbial community structure away from the seeding community and towards the calculated metacommunity. Future research should include studies that target microbial growth and activity.

## Supplementary Information

**Additional file 1: Figure S1.** Gap statistic analysis (top) and Cluster dendogram (bottom) of subsetted samples. Gap statistic analysis was used to derive the appropriate number of sample groups and the cluster dendogram was used to validate the samples in each group. **Figure S2.** Relationship between estimated abundance of *Pseudomonas* and estimated total cell abundance. No significant correlation was observed, *r*^2^=0.10. Values are in 16S rRNA copies.m2 (corrected for snow mass loading per sample). **Figure S3.** Scatterplot matrix showing histograms, kernel density overlays, absolute correlations and significance asterisks (*p*<0.05*, *p*<0.01**, *p*<0.001***), for the relationships between immigration, abundance and diversity and selected parameters including pIN (ice nucleation), Cl^**−**^, NO_3_-, POC, formic and acetic acid).

**Additional file 2: Supplementary Tables**.

**Additional file 3.**

## Data Availability

The datasets generated during and/or analyzed during the current study (raw data) and codes used for macroecology and 16 rRNA amplicon analysis are available at: 10.5281/zenodo.7398390 All data generated or analyzed during this study are included in this published article and its supplementary information files.
