# Supplementary material for: Selection processes of Arctic seasonal glacier snowpack bacterial communities
Source: Microbiome. 2023 Mar 2;11:35. doi: 10.1186/s40168-023-01473-6 (PMC9979512; doi:10.1186/s40168-023-01473-6)

Supplementary Information Figures


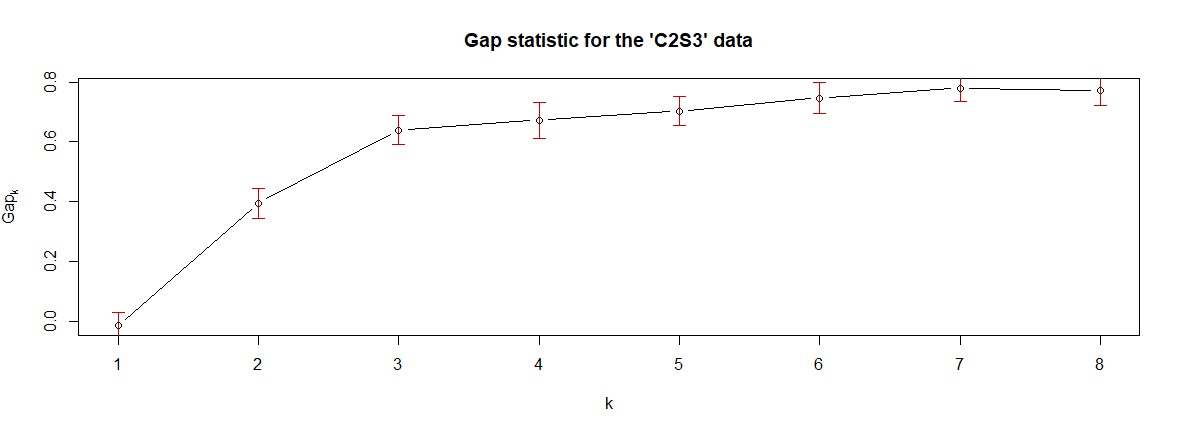


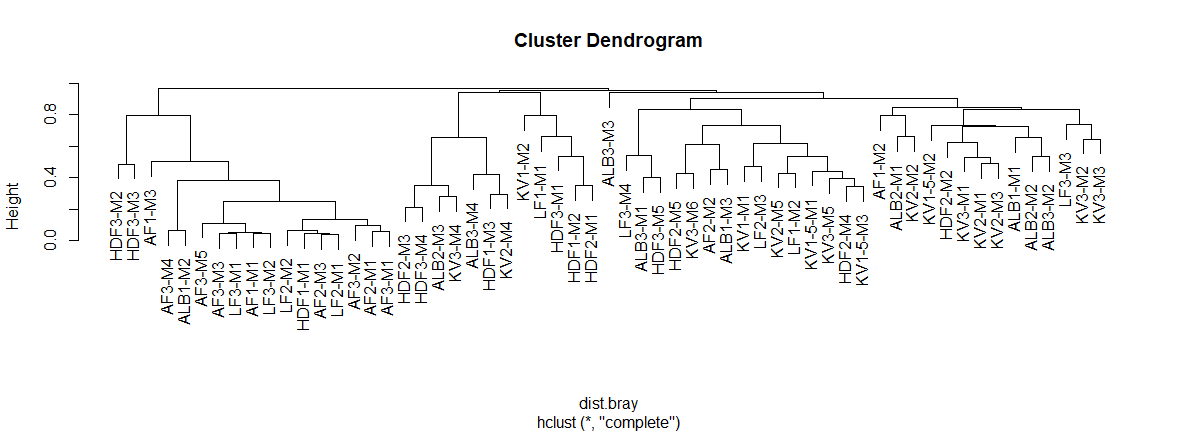


SI Figure 1: Gap statistic analysis (top) and Cluster dendogram (bottom) of subsetted samples. Gap statistic analysis was used to derive the appropriate number of sample groups and the cluster dendogram was used to validate the samples in each group.


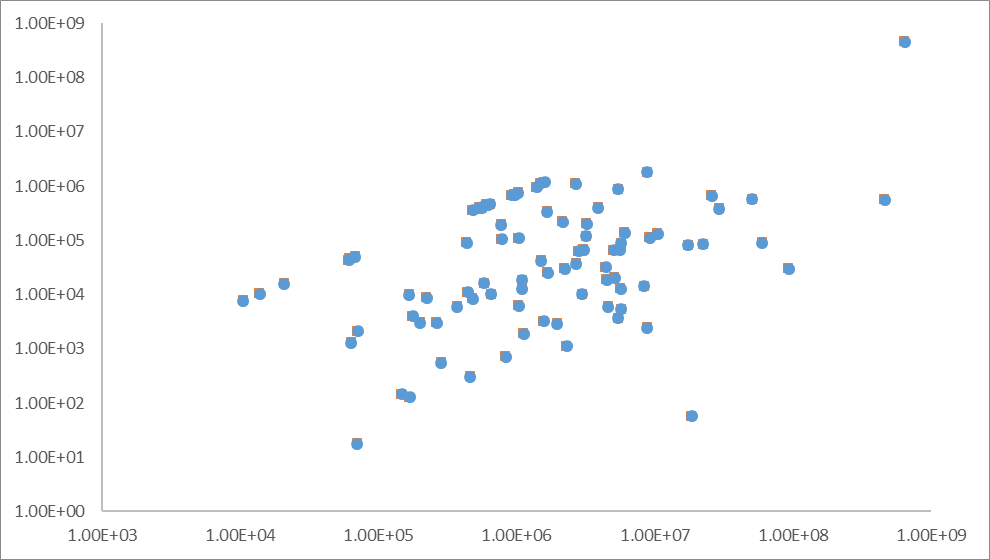


Estimated Pseudomonas abundance

Total Estimated bacterial abundance

SI Figure 2: Relationship between estimated abundance of *Pseudomonas* and estimated total cell abundance. No significant correlation was observed, r^2^=0.10. Values are in 16S rRNA copies.m2 (corrected for snow mass loading per sample).

# SI Figure 3: Scatterplot matrix showing histograms, kernel density overlays, absolute correlations and significance asterisks (p<0.05*, p<0.01**, p<0.001***), for the relationships between immigration, abundance and diversity and selected parameters including pIN (ice nucleation), Cl^-^, NO_3_-, POC, formic and acetic acid).


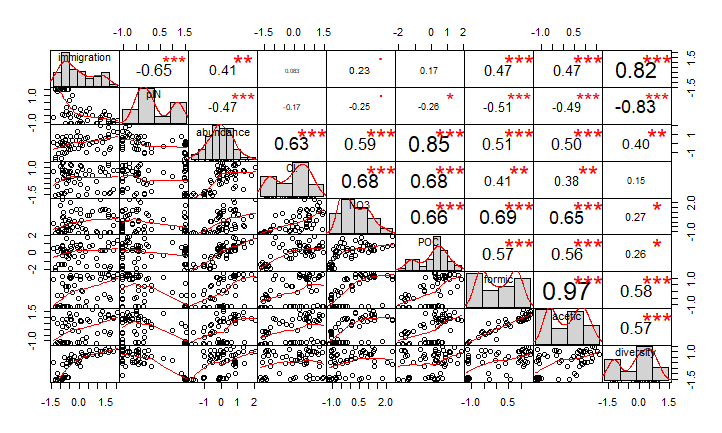

Supplement: Supplementary file 2 — Additional file 1: Figure S1. Gap statistic analysis (top) and Cluster dendogram (bottom) of subsetted samples. Gap statistic analysis was used to derive the appropriate number of sample groups and the cluster dendogram was used to validate the samples in each group. Figure S2. Relationship between estimated abundance of Pseudomonas and estimated total cell abundance. No significant correlation was observed, r2=0.10. Values are in 16S rRNA copies.m2 (corrected for snow mass loading per sample). Figure S3. Scatterplot matrix showing histograms, kernel density overlays, absolute correlations and significance asterisks (p<0.05*, p<0.01**, p<0.001***), for the relationships between immigration, abundance and diversity and selected parameters including pIN (ice nucleation), Cl−, NO3-, POC, formic and acetic acid). [file 40168_2023_1473_MOESM1_ESM.docx]
